# Nurses’ perception of the impact of professional development sessions on their pre-hospital clinical practice with trauma victims

**DOI:** 10.3389/fpubh.2024.1365509

**Published:** 2024-04-22

**Authors:** Mauro Mota, Regina Pires, Madalena Cunha, Margarida Reis Santos

**Affiliations:** ^1^Health School of the Polytechnic University of Viseu, Viseu, Portugal; ^2^UICISA: E/ESEnfC—Cluster at the Health School of Polytechnic University of Viseu, Viseu, Portugal; ^3^CINTESIS—Center for Health Technology and Services Research, University of Porto, Porto, Portugal; ^4^Nursing School of Porto, Porto, Portugal

**Keywords:** continuing nursing education, prehospital care, nursing education research, trauma, pre-hospital nurses

## Abstract

**Background:**

Continuing education is important for the quality of clinical practice because it complements it and focuses primarily on producing qualified pre-hospital nurses with operationally defined competence in nursing standards. The objective of this study was to assess pre-hospital nurses’ opinion of the impact of professional development sessions on their clinical practice.

**Method:**

A descriptive and quantitative study was carried out involving Portuguese pre-hospital nurses. Six professional development sessions were presented in 2020 to pre-hospital registered nurses in four of Portugal’s main cities. To collect the data, at the end of each session, we apply a questionnaire designed specifically for this study. This data collection instrument consists of 11 questions, six designed to evaluate the session and five designed to evaluate the trainer responsible for the session. A five-point Likert scale was used for each question, where 1 corresponds to very dissatisfied and 5 to extremely satisfied.

**Results:**

Two hundred and two nurses, which represents 55% of all Portuguese pre-hospital nurses, took part in the assessment of the professional development sessions. The nurses were from the Northern region of Portugal (51%; *n* = 102), the Centre region (29%; *n* = 59) and the Southern region of Portugal (20%; *n* = 41). Nurses found the session extremely satisfactory. All the assessment scores ranged between 4.4 and 4.7 points, on a scale of 1 to 5. 76.2% of the participants considered that the knowledge acquired could have a major impact [score = 5] on their future clinical practice. The majority of pre-hospital nurses (96.5%) felt that the session could have a major impact [score = 5; 76.2%, *n* = 154] or a very important impact [score = 4; 20.3%, *n* = 41] on their clinical practice.

**Conclusion:**

The professional development sessions provide pre-hospital nurses with the latest research findings and the majority of nurses considered that the training had a huge impact on their clinical practice. However, it is important that future research aims to explore the cause-effect relationship between training and improved clinical practice.

## 1 Background

Trauma is a leading cause of death, with more than five million deaths worldwide each year (1). This reality has led to the development of important evidence-based guidelines for the acute management of trauma patients over the past 15 years (2). Nursing care is both central and essential to the administration of high-quality care in different health care settings (3). Nursing as a professional discipline must be dynamic, capable of responding to the changing needs of individuals, societies and health systems. Only this way will it be possible to provide a knowledgeable service to a society (4). Nursing education complements nursing practice because is primarily centered on producing skilled nurses, with competence operationally defined in nursing standards (5). Educational preparation of nurses is required to provide congruent care, and nurses should be offered both formal education and clinical training, as well as continuing education (6).

In mainland Portugal, the National Institute of Medical Emergency (INEM) is the Ministry of Health body responsible for coordinating the Integrated Medical Emergency System. Pre-hospital assistance is provided by different health professionals, but nurses and physicians are the professionals who provide the most differentiated rescue teams. Pre-hospital nurses are exclusively team leaders in one of these teams, the Immediate Life Support Ambulances (ASIV). The work of nurses in the pre-hospital field in Portugal is regulated by therapeutic protocols that determine a significant part of their interventions (7).

Educational interventions, such as professional nursing education and structured discussions about clinical practice, require an active process of engagement whereby personal perspectives are shared, openly discussed and debated to achieve new learning (8). When nurses have opportunities to discuss, assess, and develop shared disciplinary and personal-professional positions, values, and procedures with others, their understanding of their practice can be greatly improved (9).

As far as the reality of pre-hospital nursing is concerned, there is a clear need to carry out further studies that will prove the importance and impact of nursing professional education in prehospital nurses’ clinical practice (10). Pre-hospital nursing must be based on care during the healing/health process, prioritizing the safety of health care. In this sense, it is necessary to develop, promote and stimulate research and professional training in the field of emergency care, which will enhance all of its assistance (11).

As such, we propose that the professional sessions developed for pre-hospital nurses contribute to deepening and updating knowledge about the reality of their practice.

The objective of this study is to assess pre-hospital nurses’ opinion of the impact of professional development sessions on their clinical practice.

## 2 Material and method

This study was conducted in accordance with the Strengthening the Reporting of Observational Studies in Epidemiology (STROBE) guidelines (12).

### 2.1 Study design

This descriptive, cross-sectional, quantitative study was conducted with Immediate Life Support Ambulances (ASIV) nurses in Portugal. It should be noted that in Portugal, nurses are part of pre-hospital rescue teams. In 2020, six professional development sessions were presented to pre-hospital registered nurses in four of Portugal’s main cities (two in Porto, two in Coimbra, one in Lisbon and one in Faro). Pre-hospital nurses attended only one of these sessions. These sessions were intended to promote the dissemination of knowledge on post-traumatic bleeding management, as it remains the leading cause of potentially preventable death among injured people (13). Acute traumatic pain management was also addressed in the sessions, as pain treatment is still undervalued in the pre-hospital setting, and less than 40% of adult patients have insufficient pain relief (14). So, the aims of the sessions were: to present and discuss “The European guideline on management of major bleeding and coagulopathy following trauma” (13); and analyse pain management interventions for trauma victims. The trainer is a nurse specialist in Medical-Surgical Nursing and a trauma specialist. He has been working in the pre-hospital environment for 10 years and considering the expertise and research he has developed in the area, he was invited to conduct the sessions. The trainer is also a professor at a Nursing University.

### 2.2 Setting

The training sessions took place at the National Institute of Medical Emergency (INEM) facilities. INEM is the Ministry of Health organization responsible for the coordination of the Integrated Medical Emergency System in mainland Portugal, providing prehospital emergency care to trauma victims and acute medical patients. It is responsible for the initial and continuing professional training of all pre-hospital providers in mainland Portugal, which includes nurses, physicians and pre-hospital emergency technicians.

Each session lasted approximately four hours, with two 15 min breaks. Participatory methodology was the method adopted during the sessions and a PowerPoint was used for this purpose. Before closing and evaluating the session, some time was taken for discussion.

“The European guideline on management of major bleeding and coagulopathy following trauma” aims to provide guidance on the management of severe bleeding and coagulopathies following traumatic injuries. These guidelines recommend that these guiding principles have to be adapted to meet individual circumstances and institutional resources. Recommendations number 1-6, 12, 13, 15, 17, 19, 38 and 39 of the “The European guideline on management of major bleeding and coagulopathy following trauma” were presented and discussed. These were the recommendations chosen since they can be replicated in pre-hospital nursing intervention (Table 1).

**Table 1:** Topics covered in the training session.

Topics	Elapsed time
Introduction and objectives	10 min
Development	
European guideline on management of major bleeding and coagulopathy following trauma:	110 min
^*^Recommendation 1—Minimal elapsed time ^*^Recommendation 2—Local bleeding management ^*^Recommendation 3—Ventilation ^*^Recommendation 4—Initial assessment ^*^Recommendation 5—Immediate intervention ^*^Recommendation 6—Further investigation ^*^Recommendation 12—Tissue oxygenation ^*^Recommendation 13—Restricted fluid volume replacement ^*^Recommendation 15—Type of fluid ^*^Recommendation 17—Temperature management ^*^Recommendation 19—Pelvic ring closure and stabilisation ^*^Recommendation 38—Guideline implementation ^*^Recommendation 39—Assessment of bleeding control and outcome	
Pharmacological and Non-pharmacological management in pain treatment	40 min
Open debate	40 min
Conclusion and evaluation of the session	10 min

^*^See Reference (13).

### 2.3 Participants

All Portuguese registered nurses who were working with ASIVs were invited to take part in the professional development sessions. Participants with the following inclusion criteria were included: (a) being a pre-hospital nurse; (b) having specific training in the treatment of trauma victims; (c) having experience in the pre-hospital care of trauma victims.

### 2.4 Data sources

To collect the data, we used a questionnaire developed specifically for this purpose by two professors from two different nursing universities, in collaboration with the trainer who conducted the session. This data collection tool comprised 11 questions, six designed to assess the session, and five to assess the trainer responsible for the session. In this questionnaire, pre-hospital nurses assessed the impact of professional development sessions on their clinical practice. Before applying the questionnaires, a pilot process was carried out with two nurses and two invited academics to identify the consistency of the scale. No changes were necessary. To answer questions related to the session and to the performance of the trainer a five points Likert-type scale was used. In this scale, 1 corresponds to very dissatisfied and 5 to extremely satisfied.

This study was approved by the INEM.

### 2.5 Data collection

At the beginning of each session, the trainer clarified the purpose and procedures of this study. The nurses were informed that they had the right to refuse to participate in the study. To protect the participants, a request for non-identifying information was made. At the end of session, the participants completed the assessment questionnaire. The data collection tool was handed out to the participants once the professional development session was over.

### 2.6 Data analysis

Descriptive statistics were used to carry out the data analysis, and statistical treatment was conducted using the version 24 for Windows of the Statistical Package for the Social Sciences (SPSS).

## 3 Results

Two hundred and two nurses, which represents 55% of all pre-hospital nurses working on ASIV, took part in the assessment of the professional development sessions. The nurses were from the Northern region of Portugal (51%; *n* = 102), the Centre region (29%; *n* = 59) and the Southern region of Portugal (20%; *n* = 41).

Nurses found the session extremely satisfactory. All the assessment scores ranged between 4.4 and 4.7 points, on a scale of 1 to 5. Nurses considered that the knowledge gained will have a huge impact on their future clinical practice (MD = 4.7), 76.2% (*n* = 154) of the participants showed they were highly satisfied (score = 5), and 66.3% (*n* = 134) reported that the session fully met their needs (score = 5). Most of the nurses (95.5%; *n* = 193) who participated in the sessions found the contents addressed extremely satisfactory [score = 5; 66.3%; *n* = 134] or very satisfactory [score = 4; 29.2%, *n* = 59]. Many nurses (96.5%; *n* = 195) considered that the session had a huge impact [score = 5; 76.2%, *n* = 154] or a very important impact [score = 4; 20.3%, *n* = 41] on their clinical practice. In most cases (13.4%, *n* = 27), the item that scored below 4 was “Was the duration of the session adequate?” and the item that got less ratings below 4 was “Were the contents addressed pertinent?” (Table 2).

**Table 2:** Assessment of the professional development sessions.

Questions	Score *n* (%)					MD	SD
	1	2	3	4	5		
Were the contents addressed pertinent?	0 (0)	0 (0)	2 (1)	48 (23.8)	152 (75.2)	4.7	0.46
Was the methodology used appropriate?	0 (0)	0 (0)	3 (1.5)	71 (35.1)	128 (63.4)	4.6	0.52
Was the management of resources adequate?	1 (0.5)	0 (0)	5 (2.5)	74 (36.6)	122 (60.4)	4.6	0.6
Was the duration of the session adequate?	1 (0.5)	4 (2)	22 (10.9)	61 (30.2)	114 (56.4)	4.4	0.8
Did the content of the session meet your needs?	0 (0)	2 (1)	7 (3.5)	59 (29.2)	134 (66.3)	4.6	0.61
Will the knowledge obtained have an impact on your future clinical practice?	0 (0)	1 (0.5)	6 (3)	41 (20.3)	154 (76.2)	4.7	0.54

MD, median; SD, standard deviation; *n*, number of nurses; %, percentage.

The way the trainer organized the session was also found extremely satisfactory. The assessment scores ranged between 4.8 and 4.9 points on a scale of 1 to 5. Most of the participants expressed maximum satisfaction regarding the clarification of doubts [score = 5; 83.7%, *n* = 169]; the trainer’s knowledge [score = 5; 93.1%, *n* = 188]; the relationship established between the trainer and the participating nurses [score = 5; 88.1% *n* = 178]. No item scored below 3 and the items with a 3 score show a mean score ranging between 0.5% (“Did the trainer show a strong knowledge of the contents addressed?” and “Has the trainer’s relationship with the group been appropriate?”) and 2% (Were you given time to discuss information and to clarify doubts?).

Most of the nurses (95.5%; *n* = 193) who participated in the sessions found the contents addressed extremely satisfactory [score = 5; 66.3%, *n* = 134] or very satisfactory [score = 4; 29.2%; *n* = 59]. The majority of them (96.5%; *n* = 195) considered that the session had an extremely important impact [score = 5; 76.2%, *n* = 154] or at least a very important impact [score = 4; 20.3% *n* = 41] on their clinical practice. Most of the participants (99.5%; *n* = 201) found that the trainer’s knowledge of the topic addressed was extremely satisfactory [score = 5; 93.1%, *n* = 188] or very satisfactory [score = 4; 6.4%, *n* = 13] (Table 3).

**Table 3:** Assessment of the *trainer*’s performance during the session.

Questions	Score *n* (%)					MD	SD
	1	2	3	4	5		
Was the trainer’s discourse clear and assertive?	0 (0)	0 (0)	2 (1)	25 (13.9)	172 (85.1)	4.8	0.39
Were the requested topics discussed with scientific accuracy?	0 (0)	0 (0)	3 (1.5)	36 (17.8)	163 (80.7)	4.8	0.44
Did the trainer show a strong knowledge of the contents addressed?	0 (0)	0 (0)	1 (0.5)	13 (6.4)	188 (93.1)	4.9	0.28
Were you given time to discuss information and to clarify doubts?	0 (0)	0 (0)	4 (2)	29 (14.4)	169 (83.7)	4.8	0.45
Has the trainer’s relationship with the group been appropriate?	0 (0)	0 (0)	1 (0.5)	23 (11.4)	178 (88.1)	4.9	0.35

MD, median; SD, standard deviation; *n*, number of nurses; %, percentage.

## 4 Discussion

Knowledge translation between academics and users accelerates the capture of the benefits of evidence-based practice through improved health, more effective services and products and a strengthened health care system (15). The majority of the nurses who took part in the sessions found the issues addressed extremely satisfactory or very satisfactory and 96.5% (*n* = 195) considered that the session was extremely important or very important for their clinical practice. The aim of the professional sessions is to promote the thinking capacity of the professional and develop their learning autonomy, always taking into account ethical concerns and the awareness of one’s ability to transform reality (16).

The assessment of the trainer’s performance during the sessions was highly valued by the participating nurses. This result highlights the importance of the sharing of research developments in the dissemination of knowledge and the improvement of practices. The combination of theory and practice is the greatest advantage of this educational initiative (17). For most of the participants, the level of knowledge demonstrated by the trainer of the topic addressed was extremely satisfactory or very satisfactory. The relationship of mutual trust established between all the parties involved makes for greater levels of interest and confidence between them. To encourage behaviour changes, to implement the desired changes into clinical practice and to reinforce progress are fundamental structural foundations for the global development of Nursing as a Science.

The development of care depends on the development of the right skills and on one’s decision-making capacity and this clearly requires greater knowledge. Evidence-based practice is fundamental in improving healthcare quality (18). The transfer of this knowledge to clinical reality may be responsible for improving assistance and increasing the overall quality of healthcare provided. There is a significant delay between the production of research results and their incorporation into clinical practice. The development of research is mainly carried out by universities and health institutions have an organizational culture that makes it difficult to transfer the knowledge produced into clinical practice (19). All teaching practice should be focused on the use of reflective and transformative educational strategies and methodologies (17).

We believe that including nurses in training sessions where results of scientific research and practice guidelines are presented will increase their interest in science, promote evidence-based practice and improve the quality of care. The trainer needs to mobilize his knowledge according to the needs of the group, using teaching strategies that will help him deliver his best teaching and that will allow the personal growth, personally as well as collectively, of both trainers and students (20). Educational practice is based on the relationship between the content of learning, the subject who learns and the trainer, following the socio-interactionist theory of education (21). The close relationship between the research team and the nurses responsible for data collection must also be strengthened, so that all parties understand the benefit of this collaboration. It will also be important to integrate theoretical and practical teaching, and for this a space for discussion must be opened (20). Thus, 94.1% of the participating nurses (*n* = 190) considered that during the research they were given excellent conditions to clarify doubts and discuss therapeutic measures to be implemented in the treatment of trauma victims. Therefore, cooperation between academia and clinical practice needs to be in close consonance with its own pedagogical projects, promoting important and productive reflective moments for professionals (16).

### 4.1 Implications for practice

The continuous professional development sessions on specific topics were very well received by nurses. Pre-hospital nurses valued the training sessions and felt that the content contributed to their professional development. Training sessions should be carried out considering the time of the session and contain information that responds to the specific needs of pre-hospital nurses. The professional training courses seem to have responded to the needs felt by the pre-hospital nurses and the organization itself with regard to assisting trauma victims with major bleeding.

### 4.2 Limitations

This study has some limitations. First, it is not possible to establish a cause-effect relationship between the training sessions and the improvement of clinical practice since these results are only the result of the evaluation of nurses’ opinions. Second, the impossibility of evaluating the effects of the training does not allow us to infer whether the planning of the training was optimal and at what points it could be optimized. Finally, the conclusions of this research should be interpreted considering the nurses’ opinion at the end of the session, i.e., it would be important to understand their opinion after having new contacts with new trauma emergencies.

## 5 Conclusion

In this study, we examined nurses’ opinions about training sessions and their implications for clinical learning. The nurses attached great importance to the inclusion of an extended discussion period in the training session. They considered that this strategy allowed a greater involvement in the training and allowed them to deepen their knowledge. They also recognized the appropriateness of the adult training strategy. The nurses assumed that the professional development sessions they attended allowed them to improve their clinical practices. The professional development sessions provide nurses with the latest research findings and they considered that the training had a huge impact on their clinical practice, which may demonstrate the need to use these trainings to maintain effectiveness and safety in the pre-hospital care provided by nurses.

## Data availability statement

The raw data supporting the conclusions of this article will be made available by the authors, without undue reservation.

## Ethics statement

This study was approved by INEM and is part of the project “Evidências para Não Arriscar MaisVidas: do pré-hospitalar ao serviço de urgência e a alta (MaisVidas),” with the reference: PROJ/UniCISE/2017/0001 and got favorable ethical approval from the Tondela Viseu Hospital Centre Ethics Committee. Written informed consent was obtained from the participants. The protection of human subjects and Institutional Review Board (IRB) approval has been secured.

## Author contributions

MM: Writing – original draft, Writing – review & editing. RP: Writing – original draft, Writing – review & editing. MC: Writing – original draft, Writing – review & editing. MS: Writing – original draft, Writing – review & editing.
